# Assessing the Impact of Air Quality and Socioeconomic Conditions on Respiratory Disease Incidence

**DOI:** 10.3390/tropicalmed10020056

**Published:** 2025-02-17

**Authors:** Mustfa Faisal Alkhanani

**Affiliations:** Biology Department, College of Science, University of Hafr Al Batin, P.O. Box 1803, Hafr Al Batin 39524, Saudi Arabia; mfalkhanani@uhb.edu.sa

**Keywords:** air quality, respiratory disease, public health, nitrogen dioxide, pulmonary disease, asthma, tuberculosis

## Abstract

**Background and Objective:** Air pollution poses significant risks to global public health and has well-established links to respiratory diseases. This study investigates the associations between air pollution markers—Air Quality Index (AQI), ambient ozone, and nitrogen dioxide (NO_2_)—and the incidence of chronic obstructive pulmonary disease (COPD), asthma, and tuberculosis. It also examines how socioeconomic factors such as gross domestic product (GDP) per capita, tobacco prevalence, and healthcare expenditure influence these relationships. This study includes data from 27 countries, thereby offering a global perspective to inform public health interventions and policy reforms. **Methods:** Data on average air pollution levels, respiratory disease incidence, and socioeconomic factors were collected from publicly available sources spanning four years. The 27 countries included in the study were selected to represent a broad range of pollution levels, income brackets, and geographical regions. Statistical analyses were performed using Python 3.12.0 to explore the relationships between these variables. **Key Findings:** AQI and NO_2_ levels were significantly associated with increased incidences of COPD and tuberculosis, with rates rising especially during periods of heightened pollution. Conversely, ambient ozone exhibited inconsistent relationships with respiratory diseases, heavily influenced by socioeconomic factors. Higher GDP per capita and healthcare expenditure were linked to improved management of infectious diseases like tuberculosis, though they also corresponded with higher reporting of chronic conditions such as COPD. Tobacco smoking emerged as a critical risk factor for COPD across all regions. **Conclusions:** This study underscores the strong associations between air pollutants and respiratory diseases, particularly tuberculosis and COPD, with socioeconomic factors significantly influencing these relationships. Reducing air pollution and improving healthcare systems, particularly in low-income regions, are essential to mitigating the global burden of respiratory diseases.

## 1. Introduction

Air pollution is a critical global health issue, contributing to an estimated 7 million premature deaths annually, with the majority resulting from respiratory and cardiovascular diseases [1]. This underscores the severe health consequences of prolonged exposure to harmful pollutants such as fine particulate matter (PM2.5), nitrogen dioxide (NO_2_), and ozone. Exposure to these pollutants can lead to or exacerbate respiratory conditions such as asthma and chronic obstructive pulmonary disease (COPD), and infectious diseases like tuberculosis. The Air Quality Index (AQI), a comprehensive metric encompassing multiple pollutants, has become a critical tool in assessing air quality and its associated health risks. Alongside AQI, specific measures of ambient ozone and NO_2_ levels are increasingly employed in research to understand the complex dynamics between pollution exposure and respiratory disease incidence [2].

Extensive research has shown that air pollution significantly affects respiratory health, particularly chronic conditions such as asthma and COPD. Metrics like the Air Quality Index (AQI), ozone levels, and nitrogen dioxide (NO_2_) concentrations are widely used to study these relationships. For instance, higher AQI levels have been consistently associated with an increase in hospital visits and exacerbation of respiratory illnesses, especially in vulnerable groups such as children and the elderly [2]. Ambient ozone formed through chemical reactions between pollutants from vehicles and industrial emissions poses serious respiratory risks. Studies have shown how exposure to ozone worsens conditions such as asthma by inducing airway inflammation and oxidative stress [3,4]. Long-term exposure to high ozone levels has also been associated with a higher prevalence of COPD and other chronic respiratory disorders, particularly in regions with significant industrial pollution [5,6,7]. Similarly, nitrogen dioxide (NO_2_), which is primarily emitted from motor vehicles and industrial activities, is another pollutant of concern. Prolonged exposure to NO_2_ has been shown to impair lung function, elevate the risk of respiratory infections, and accelerate the progression of diseases such as COPD [8,9]. Although the link between NO_2_ and COPD is well established, its association with other diseases like asthma and tuberculosis remains underexplored, warranting further investigation.

The relationship between air pollution and respiratory diseases is further influenced by socioeconomic factors such as GDP per capita, smoking prevalence, and healthcare expenditure [10,11]. Higher-income countries with advanced healthcare systems may be better equipped to mitigate the health impacts of pollution through advanced medical care and public health interventions. Conversely, lower-income nations face the compounded challenges of high pollution levels and inadequate healthcare infrastructure, exacerbating the burden of respiratory diseases [12,13]. GDP per capita is often used as a proxy for a country’s economic status and healthcare infrastructure, since wealthier nations have been observed to have lower rates of tuberculosis and other infectious diseases due to better access to healthcare and improved living conditions [14]. Asthma prevalence however remains high in high-income countries, suggesting that indoor pollution and lifestyle factors such as urbanization may play a more significant role [15,16].

Tobacco smoking is a well-established risk factor for respiratory diseases, particularly COPD and tuberculosis [17]. Smoking exacerbates the effects of air pollution by weakening the respiratory system, making individuals more susceptible to the harmful effects of pollutants like NO_2_ and ozone [18]. Studies have shown that countries with high smoking prevalence and elevated pollution levels have a significantly higher incidence of respiratory diseases, stressing the need for comprehensive public health strategies that address both smoking cessation and pollution control [19,20]. Healthcare expenditure is another critical factor influencing the detection and management of respiratory diseases. Countries with higher healthcare spending tend to have more accurate diagnoses and better management of chronic conditions like asthma and COPD. In contrast, low-income countries with limited healthcare access face higher burdens of infectious diseases such as tuberculosis, particularly in regions with high pollution levels [21].

While existing studies have provided valuable insights into the relationships between air pollution and respiratory diseases, several gaps remain. Most studies have limited applicability because they focus on single pollutants or localized regions. Moreover, few studies have incorporated socioeconomic factors such as GDP, smoking rates, and healthcare expenditure into their analyses despite the clear influence these factors have on health outcomes. This research addresses these gaps by analyzing data from 27 countries over a four-year period, using a comprehensive approach that includes multiple pollutants and socioeconomic factors to give a more holistic exploration of the influence of air pollution on respiratory health. The insights gained aim to guide targeted public health initiatives designed to mitigate the global impact of respiratory diseases.

This study seeks to investigate the associations between AQI, ambient ozone levels, and NO_2_ concentrations with the incidence of asthma, COPD, and tuberculosis across 27 countries over a four-year period (2018–2021). Specifically, this research aims to answer the following questions:

1. What are the associations between the AQI, ambient ozone, and NO_2_ concentrations, and the incidence rates of asthma, COPD, and tuberculosis?
2. How do socioeconomic factors such as GDP per capita, tobacco smoking prevalence, and healthcare expenditure influence these associations?
3. How do periods of high and low air pollution affect the incidence of respiratory diseases such as tuberculosis, COPD, and asthma?

The hypothesis of this study is that higher levels of air pollution, as measured by the Air Quality Index (AQI), ozone, and NO_2_, are associated with increased incidence rates of asthma, COPD, and tuberculosis, especially in countries with lower GDP per capita, higher smoking rates, and lower healthcare expenditure. Controlling for these socioeconomic factors will clarify how environmental and economic conditions interact to affect respiratory health. Additionally, it is hypothesized that there will be significant differences in disease incidence between high- and low-pollution periods, with higher rates of respiratory diseases, particularly tuberculosis and COPD, occurring during high-pollution periods.

## 2. Methodology

This section presents the study design which details the relationship between respiratory disease outcomes and air pollution across 27 countries over a four-year period (2018–2021). Sources of data, statistical analysis, ethical considerations, and potential bias in data sources are also presented in this section.

### 2.1. Study Design

This study utilized a cross-sectional design to investigate the relationship between air pollution and respiratory disease outcomes across 27 countries (Appendix A) over four years (2018–2021). Although data were collected over a four-year period, the analysis was conducted at a single point in time by using average values over the study period. This approach ensures that the study remains cross-sectional, as it examines associations between air pollution and respiratory health without assessing temporal trends.

This design allowed for the analysis of multiple variables simultaneously, providing a comprehensive view of both global trends and country-specific variations. The 27 countries included in this study were carefully selected to ensure geographic diversity, variation in pollution levels, and socioeconomic representation. To achieve this, we chose six countries from each major continent (Asia, Africa, Europe, North America, and South America) and two from Australia, capturing a broad spectrum of air pollution exposure, income brackets, and healthcare infrastructures. This approach allowed us to explore how the relationships between air pollution and respiratory diseases differ across regions with distinct environmental and economic conditions.

Countries with incomplete or inconsistent data on key variables were excluded to maintain the integrity and comparability of our analysis. By ensuring that only countries with reliable data were included, we strengthened the validity of our findings while preserving the study’s global perspective. Including a mix of high-income and low-income countries ensures the study captured diverse settings, shedding light on how economic and environmental disparities impact health outcomes.

### 2.2. Variable Selection

The Air Quality Index (AQI), ozone, and nitrogen dioxide (NO_2_) were selected as primary air pollution indicators due to their well-established roles in influencing respiratory health [22,23,24]. The AQI is a composite measure that reflects overall air quality, including concentrations of various pollutants like particulate matter (PM2.5), carbon monoxide, and sulfur dioxide, making it a comprehensive indicator of general air pollution levels. Ozone and NO_2_, on the other hand, are two key pollutants that have been extensively studied for their direct effects on lung function and respiratory diseases [3,5,25,26,27]. Ozone, a reactive gas formed by the interaction of sunlight with pollutants, is known to cause airway inflammation, while NO_2_, primarily generated from vehicular and industrial emissions, is a well-documented contributor to chronic respiratory conditions such as asthma and COPD [27]. By including these variables, this study aimed to capture a broad spectrum of air pollution’s impact on respiratory health, as well as the differential effects of individual pollutants. The AQI is calculated using the following equation:

$$\mathrm{AQI}=\frac{\left(I_{high}-I_{low}\right)}{{(C}_{high}-C_{low})}\times (C-C_{low})+I_{low}$$

where C = observed concentration of the pollutant.

$C_{low}$ = lower bound of the AQI category for the given pollutant concentration.

$C_{high}$ = upper bound of the AQI category for the given pollutant concentration.

$I_{low}$ = lower bound of the AQI range for the pollutant category.

$I_{high}$ = upper bound of the AQI range for the pollutant category.

In addition to air pollution variables, socioeconomic factors were included to account for their potential confounding effects and to explore how they may modify the relationship between air pollution and respiratory disease. Tobacco use is a well-known risk factor for respiratory diseases [19,28], particularly COPD [17], and its prevalence can act as an important confounder in studies examining air pollution and respiratory outcomes. As smoking is a major cause of lung damage, the study controlled for this factor to isolate the independent effects of air pollution. Similarly, GDP per capita and healthcare expenditure were included as proxies for a country’s level of economic development and healthcare infrastructure. Higher GDP often correlates with better healthcare access [29], advanced medical technologies, and improved public health policies, which can influence the detection, management, and outcomes of respiratory diseases. Conversely, healthcare expenditure can directly affect the quality and availability of health services, such as respiratory disease treatments and air quality-monitoring programs. Both of these variables are crucial in understanding the broader social and economic context in which air pollution affects public health, as they may influence not only disease prevalence but also the capacity of nations to address and mitigate the health impacts of air pollution.

### 2.3. Source of Data

To ensure the accuracy and credibility of this study, data were obtained exclusively from reputable databases to ensure accuracy and credibility.

1. Air Pollution Data: AQI values were obtained from the IQAir World Air Quality (https://www.iqair.com/world-most-polluted-countries (accessed on 20 October 2024)) which compiles pollution data from governments and independent monitoring stations globally. Since this study used secondary AQI data, AQI value was not directly calculated but relied on the estimates provided by IQAir. These values are derived from ground-based air quality-monitoring stations, satellite observations, and machine learning models that integrate meteorological data to improve accuracy in areas with limited ground monitoring. While we do not have access to IQAir’s proprietary equations, their data are consistent with international AQI calculation standards, making them a reliable source for this study (https://www.iqair.com/ca/newsroom/airvisual_vs_nowcast?srsltid=AfmBOop4_mw1JwaOz-dkunr_PeMF2zJ8igqOd2D--jAFSoSR3nbN6Odq (accessed on 20 October 2024)). Ambient ozone and NO_2_ levels were sourced from the Global Burden of Disease GBD Results Tool (https://vizhub.healthdata.org/gbd-results/# (accessed on 20 October 2024)), which provides population-weighted averages, accounting for pollutant exposure across diverse demographics.
2. Health Data: The incidence rates for asthma, COPD, and tuberculosis were obtained from the GBD Results Tool (https://vizhub.healthdata.org/gbd-results/# (accessed on 20 October 2024)). These rates were standardized across countries, covering all age groups and sexes.
3. Socioeconomic Indicators: Data on GDP per capita and tobacco smoking prevalence were retrieved from the World Bank Database (https://databank.worldbank.org/source/world-development-indicators (accessed on 20 October 2024)) while healthcare expenditure statistics were sourced from the WHO Global Health Expenditure Database (https://www.who.int/data/gho/data/indicators/indicator-details/GHO/current-health-expenditure-(che)-per-capita-in-us-dollar (accessed on 20 October 2024)). These indicators provided essential context for controlling confounding effects on health outcomes.

Countries were selected based on data availability and completeness, ensuring the inclusion of a diverse range of pollution levels, income brackets, and healthcare infrastructures. The inclusion criteria ensured that the findings could be generalized to various global contexts.

### 2.4. Statistical Analysis

All statistical analyses were conducted using Python 3.12.0. Libraries such as pandas for data manipulation, statsmodels for multivariate regression analysis, and scipy for Pearson correlation analysis were used. The level of significance was defined as *p*-value ≤ 0.05. The analysis involved exploring the relationships between air pollution indicators (AQI, ozone, NO_2_) and disease incidences (asthma, COPD, tuberculosis) while adjusting for potential confounders (GDP per capita, tobacco use, and health expenditure). Visualizations were generated using matplotlib 3.7.0 to present regression results and statistical associations.

Standard multivariate linear regression models were used to assess the impact of each air pollution marker (AQI, ozone, NO_2_) on the incidence of asthma, COPD, and tuberculosis. Each model was adjusted for the confounders (GDP per capita, tobacco use, health expenditure) to isolate the independent effect of air pollution on respiratory health outcomes. All predictor variables (air pollution markers and confounders) were entered into the model simultaneously. Variance Inflation Factor analysis confirmed no significant multicollinearity.

The following general model structure was applied for each disease outcome:

Disease Incidence*_i_* = β_0_ + β_1_AQI*_i_* + β_2_O3*_i_* + β_3_NO2*_i_* + β_4_GDP*_i_* + β_5_Tobacco Use*_i_* + β_6_Health Expenditure*_i_* + ϵ*_i_*

- β_1_, β_2_, and β_3_ represent the coefficients for air pollution markers.
- β_4_, β_5_, and β_6_ represent the coefficients for confounders.
- *_i_* represents each country.

One-way ANOVA tests were conducted to determine whether respiratory disease incidence rates differed significantly between high- and low-pollution periods. Countries were stratified into high- and low-pollution groups based on the median air pollution markers of AQI, ozone and NO_2_, and the differences in asthma, COPD, and tuberculosis rates between these periods were tested for statistical significance.

### 2.5. Ethical Considerations

This study utilized publicly accessible data and no human subjects. There were no ethical concerns related to the use of human subjects. All datasets were de-identified and reported at the national level to ensure the absence of personal or sensitive information. The study complied fully with ethical standards for secondary data usage without requiring formal ethical approval as there was no direct interaction with individuals.

### 2.6. Potential Bias in Data Sources

**Figure 1:**
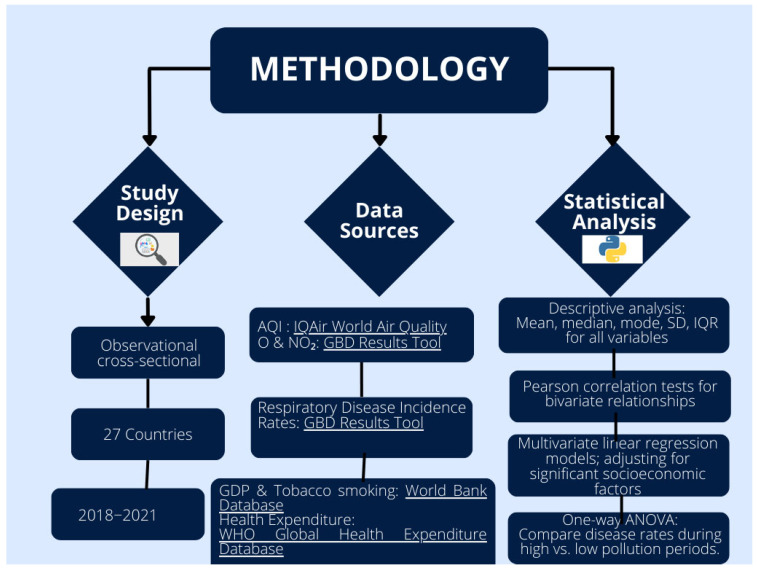
Flowchart summarizing study methodology.

The data for this study were obtained from reputable and globally recognized databases. However, it is essential to consider the potential for biases arising from differences in how countries report air pollution and health statistics. Variations in air quality-monitoring systems, accuracy of measurements, and frequency of data collection may lead to inconsistencies in the reported levels of AQI, ozone, and nitrogen dioxide. Similarly, health reporting systems differ in reliability, and underreporting of respiratory diseases is more common in countries with limited healthcare infrastructure. Such disparities may impact the findings and limit their generalizability. In addition to the known biases, it is possible that other unaccounted factors, such as differences in environmental policies, urbanization levels, and access to healthcare, could further influence the observed relationships between pollution and respiratory health outcomes. These challenges highlight the need for careful interpretation of cross-country comparisons, as differences in data quality could influence the observed relationships between pollution and respiratory health. Future research should aim to resolve these discrepancies by obtaining more detailed and standardized data across regions. Figure 1 summarizes and presents the entire research methodology in a flowchart.

## 3. Results

This section presents a statistical summary of the dataset. Correlation analysis, multivariate regression, and limitations of the study are presented in this section.

### 3.1. Summary Statistics

Table 1 below provides the descriptive statistics of the key variables. Measures of central tendency (mean, median) and dispersion (standard deviation, interquartile range) were used to summarize the data. AQI values vary widely (5–97, mean = 21.68, Std Dev = 19.09), reflecting differences in pollution exposure, while ozone and NO_2_ concentrations also show significant variability (max = 49.16 ppb and 35.33 ppb, respectively). Asthma has the highest mean prevalence (558.40 per 100k), followed by COPD (225.99 per 100k) and tuberculosis (79.82 per 100k), with TB showing high variability (Std Dev = 112.93, max = 452.63 per 100k). Socioeconomic disparities are evident, with GDP per capita ranging from USD 758.30 to USD 71,056.00 and healthcare expenditure from USD 23.85 to USD 12,051.10, suggesting that economic inequality may influence how countries manage respiratory diseases and pollution exposure.

### 3.2. Correlation Analysis

#### 3.2.1. Across All Countries

Table 2 displays the outcomes of the Pearson correlation study about the relationship between pollutants and the incidence of respiratory diseases across the sample countries. The relationship between AQI and respiratory diseases showed significant negative correlations with both asthma (r = −0.320, *p* < 0.001) and COPD (r = −0.340, *p* < 0.001), while a significant positive association was observed with tuberculosis incidence (r = 0.377, *p* < 0.001). Similarly, ozone levels negatively correlated with asthma (r = −0.257, *p* = 0.007) and COPD (r = −0.269, *p* = 0.005) while showing a strong positive association with tuberculosis (r = 0.377, *p* < 0.001). NO_2_ followed a different pattern, with significant negative correlations with asthma (r = −0.265, *p* = 0.005) and COPD (r = −0.323, *p* < 0.001), and a strong negative association with tuberculosis (r = −0.404, *p* < 0.001). These findings suggest differential effects of pollutants on chronic versus infectious respiratory diseases, possibly influenced by regional pollution control policies, immune response mechanisms, and socioeconomic conditions.

**Figure 2:**
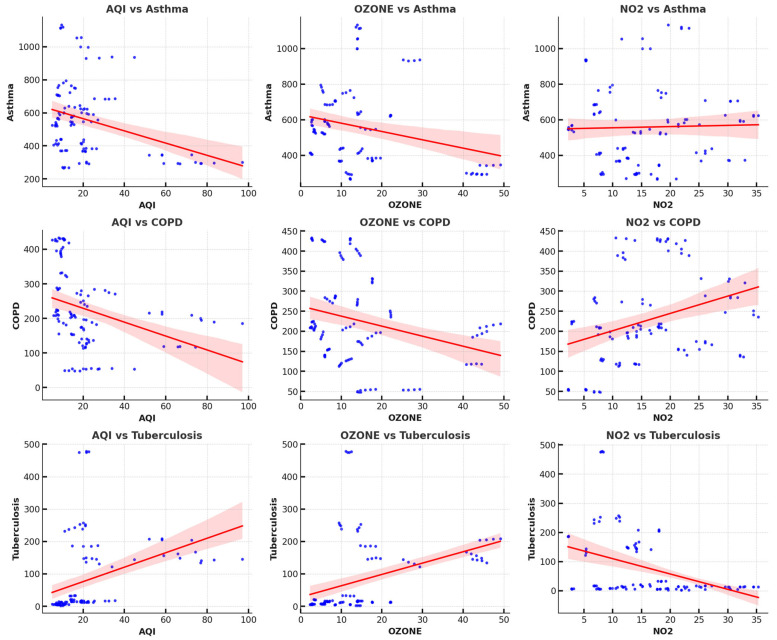
Scatter plot showing correlation between air pollutants and respiratory disease incidence.

Figure 2 depicts the scatter plots that reveal the relationships between the AQI, ozone, and NO_2_ levels and respiratory diseases (asthma, COPD, and tuberculosis). The scatter plots suggest distinct patterns for each disease. Asthma cases are widely distributed across pollutant levels, indicating multiple influencing factors beyond air pollution. Tuberculosis cases, on the other hand, show a concentration at lower pollutant levels and a weaker correlation with air quality indicators, reflecting their infectious nature rather than being predominantly pollution-driven.

In contrast, COPD exhibits a clearer trend across pollutants, particularly ozone and NO_2_, with higher pollutant levels correlating with increased COPD cases. This aligns with known biological mechanisms where these pollutants exacerbate airway inflammation and chronic respiratory damage. The scatter plots for COPD are less dispersed compared to those for asthma, suggesting that pollution may have a more direct impact on COPD than on asthma. Tuberculosis remains relatively unaffected by pollutant variations, reflecting its primary dependence on infectious and socioeconomic factors rather than environmental pollutants.

The scatter plots indicate that air pollutants like ozone and NO_2_ are more strongly associated with COPD than asthma or tuberculosis. Asthma appears to be influenced by a broader range of factors, while tuberculosis shows minimal correlation with pollution. These observations highlight the need for targeted strategies in managing respiratory diseases based on their distinct environmental and biological drivers.

#### 3.2.2. Country-by-Country Correlation

Table 3 presents the results of the country-specific Pearson correlation analysis, showing the significant relationships between air pollution markers—AQI, ozone, and NO_2_—and the incidence of asthma, COPD, and tuberculosis. The findings reveal distinct trends across different pollutants and diseases. While some trends align with broader expectations, others suggest unique regional influences that shape these relationships.

A positive correlation between AQI and tuberculosis was found in several countries, such as Japan (r = 0.986, *p* = 0.014) and South Africa (r = 0.850, *p* = 0.038), indicating that higher AQI levels are associated with increased tuberculosis incidence. This could be due to urban crowding and prolonged exposure to fine particulate matter, which has been linked to weakened immune function. In contrast, India, despite its high pollution levels, did not exhibit a similar trend. This discrepancy may stem from differences in TB surveillance, healthcare accessibility, and the prevalence of other risk factors such as malnutrition or HIV co-infections.

Asthma correlations also varied significantly. In Costa Rica (r = 0.986, *p* = 0.014) and Portugal (r = 0.995, *p* = 0.004), the AQI was positively associated with asthma, aligning with the expectation that pollution exacerbates airway hypersensitivity. However, in Chile (r = −0.958, *p* = 0.042) and South Africa (r = −0.956, *p* = 0.044), ozone had a negative correlation with asthma, suggesting that climatic or seasonal variations may influence asthma triggers differently across regions.

The relationship between AQI and COPD varied across countries. Thailand showed a negative correlation (r = −0.990, *p* = 0.010), suggesting that as AQI levels decreased, COPD incidence also dropped. Conversely, in other countries like India, no significant relationship was detected, emphasizing regional differences in disease dynamics.

Ozone was significantly associated with COPD in multiple countries. For instance, Poland (r = 0.978, *p* = 0.022), India (r = 0.765, *p* = 0.023), and Thailand (r = 0.956, *p* = 0.044) all demonstrated a positive correlation between ozone and COPD incidence, suggesting that higher ozone levels were linked to an increase in COPD cases. However, in countries like the United States (r = −0.99, r = 0.00041), Portugal (r = −0.9902, *p* = 0.0098), Sweden (r = −0.961, *p* = 0.039) and Macedonia (r = −0.998, *p* = 0.001), ozone exhibited a negative correlation with COPD, an unexpected finding that may reflect regional differences in emission sources, healthcare interventions, or variations in population susceptibility.

A contrasting pattern emerged in Brazil. While AQI correlated positively with tuberculosis (r = 0.982, *p* = 0.018), ozone had a strong negative correlation with TB (r = −0.999, *p* < 0.001). This suggests that certain pollutants may exacerbate TB transmission, while others could play a role in reducing bacterial survival or altering transmission dynamics. A similar negative correlation between ozone and TB was found in Colombia (r = −0.999, *p* = 0.0003), Pakistan (r = −0.993, *p* = 0.007), and Nigeria (r = −0.998, *p* = 0.0016), possibly due to interactions between air quality policies and disease control efforts.

For tuberculosis, a negative correlation with ozone was observed in Poland (r = −0.987, *p* = 0.013) and India (r = −0.703, *p* = 0.045), indicating that higher ozone levels were associated with a decrease in tuberculosis incidence.

The relationship between NO_2_ and COPD was consistently positive across several countries. Poland showed a strong positive correlation (r = 0.973, *p* = 0.027), as did South Africa (r = 0.823, *p* = 0.045). These results indicate that higher NO_2_ levels are significantly associated with increased COPD incidence in these countries. A similar trend was found for tuberculosis in South Africa (r = 0.850, *p* = 0.038), where higher NO_2_ levels corresponded with greater tuberculosis incidence. In Pakistan (r = −0.954, *p* = 0.046), however, NO_2_ was negatively associated with COPD, which contradicts studies that commonly link nitrogen dioxide to lung inflammation and disease progression. This may indicate strict NO_2_ regulations or reduced exposure in areas with high COPD prevalence, warranting further investigation.

### 3.3. Socioeconomic Factors

#### 3.3.1. Across All Countries

The socioeconomic variables that were considered as potential covariates for disease incidence across all countries are summarized in Table 4 below. Pearson correlation analysis results revealed significant relationships between socioeconomic confounders and respiratory disease incidence. GDP per capita showed a strong positive association with COPD (r = 0.77, *p* < 0.001), indicating that wealthier countries report higher COPD rates, likely due to better diagnostic capabilities. Conversely, GDP was negatively associated with tuberculosis (r = −0.55, *p* < 0.001), suggesting that wealthier countries have lower rates of tuberculosis, likely due to better healthcare infrastructure. No significant correlation was found between GDP and asthma (r = 0.18, *p* = 0.12).

Tobacco use was negatively correlated with asthma incidence (r = −0.29, *p* = 0.01) but positively associated with COPD (r = 0.31, *p* = 0.005), highlighting smoking as a key risk factor for the disease. No significant correlation was found between tobacco use and tuberculosis (r = 0.01, *p* = 0.95).

Health expenditure was significantly correlated with both asthma (r = 0.26, *p* = 0.02) and COPD (r = 0.74, *p* < 0.001), suggesting that better healthcare systems are associated with increased detection and reporting of these diseases. It was negatively correlated with tuberculosis (r = −0.48, *p* < 0.001), indicating that higher health spending is linked to lower tuberculosis incidence.

In summary, higher GDP and health expenditure are associated with better outcomes for chronic diseases like COPD and lower tuberculosis rates, while tobacco use remains a significant factor for COPD. These findings highlight the importance of socioeconomic factors in shaping respiratory disease prevalence.

#### 3.3.2. Country by Country

The Pearson correlation analysis of socioeconomic factors and respiratory disease incidence revealed several significant country-specific relationships as shown in Table 5. GDP per capita demonstrated diverse associations with COPD. In countries like Turkey (r = −0.99, *p* = 0.04), Angola (r = −0.99, *p* = 0.007), and Chile (r = −0.99, *p* = 0.05), GDP per capita showed a negative correlation with COPD incidence, suggesting that wealthier nations may have better healthcare infrastructure, which could mitigate COPD risks. However, Ethiopia exhibited a positive correlation (r = 0.99, *p* = 0.01), implying that higher GDP in this country might coincide with more frequent diagnosis and reporting of COPD, possibly due to improved diagnostic capabilities and healthcare access.

When examining tuberculosis, the relationship with GDP per capita was more varied. A positive correlation was found in Australia (r = 0.99, *p* = 0.02), which could reflect ongoing challenges in controlling tuberculosis despite the country’s wealth. Conversely, in New Zealand and Bangladesh, a negative correlation was observed (r = −0.99, *p*-values of 0.001 and 0.05, respectively), suggesting that higher GDP in these countries may be linked with better tuberculosis control measures and lower incidence rates.

Tobacco consumption also had distinct effects on asthma incidence. In Australia (r = 0.99, *p* = 0.04) and New Zealand (r = 0.99, *p* = 0.03), tobacco use was positively correlated with asthma), indicating that higher tobacco consumption may exacerbate asthma cases in these nations. On the other hand, Poland (r = −0.99, *p* = 0.003) and Sweden (r = −0.99, *p* = 0.009) showed negative correlations, which could point to differences in smoking patterns, national health policies, or diagnostic practices that may reduce the impact of tobacco on asthma in these countries.

Health expenditure was positively correlated with COPD incidence in several countries, including India (r = 0.99, *p* = 0.01), Ethiopia (r = 0.99, *p* = 0.03), Thailand (r = 0.99, *p* = 0.04), and Bangladesh (r = 0.99, *p* = 0.04). This suggests that higher health expenditure is associated with increased detection and reporting of COPD cases, potentially due to enhanced healthcare services and infrastructure. Conversely, in Bangladesh, health expenditure showed a negative correlation with tuberculosis (r = −0.99, *p* = 0.02), which may indicate that increased spending on healthcare programs is more effective in reducing tuberculosis incidence in this country.

These findings emphasize the complex and context-specific role of socioeconomic factors in the incidence of respiratory diseases across different countries. While higher GDP and health expenditure appear to improve the management of chronic conditions like COPD, they may not always correlate with reduced incidence of diseases like tuberculosis, highlighting the need for tailored healthcare strategies. Additionally, the relationship between tobacco use and asthma underscores the importance of effective public health policies to curb smoking rates, especially in countries with higher tobacco consumption.

### 3.4. Multivariate Regression Analysis

The result of the multivariate regression analysis in Table 6 examined the associations between air pollution markers (AQI, ozone, and NO_2_) and the incidence of asthma, COPD, and tuberculosis, while controlling for significant covariates such as GDP per capita, smoking prevalence, and healthcare expenditure.

For asthma, the analysis revealed no significant associations with any of the pollutants. AQI showed a negative relationship with asthma (β = −4.33, *p* = 0.56), but this was not statistically significant. Similarly, ozone (β = 2.997, *p* = 0.552) and NO_2_ (β = 3.937, *p* = 0.211) exhibited positive but non-significant associations with asthma incidence.

In the case of COPD, the findings were more telling. AQI demonstrated a significant negative correlation with COPD (β = −7.34, *p* = 0.0087), suggesting that higher AQI levels are associated with an increase in COPD prevalence. This relationship is noteworthy as it highlights the impact of poor air quality on respiratory health. However, ozone (β = 2.05, *p* = 0.258) and NO_2_ (β = 1.26, *p* = 0.254) did not show significant associations with COPD incidence.

For tuberculosis, NO_2_ displayed a significant negative association with disease incidence (β = −5.10, *p* < 0.001), indicating that higher NO_2_ levels are linked to a lower incidence of tuberculosis. This finding is unexpected and warrants further investigation to explore the mechanisms behind this relationship. Ozone approached significance (β = 4.31, *p* = 0.0673), suggesting a potential positive link with tuberculosis incidence, but the result was not statistically significant. AQI, however, did not show a significant association with tuberculosis (β = 0.29, *p* = 0.907).

These results underscore the differential effects that various air pollutants have on respiratory diseases. AQI was significantly associated with COPD, indicating that poor air quality contributes to the prevalence of chronic respiratory conditions. Conversely, NO_2_ was negatively correlated with tuberculosis incidence, suggesting that higher levels of this pollutant might be associated with lower tuberculosis rates, though the underlying reasons for this remain unclear. The findings also highlight the importance of controlling for relevant covariates, such as socioeconomic factors and healthcare expenditure, to accurately assess the impacts of air pollution on respiratory diseases.

**Figure 3:**
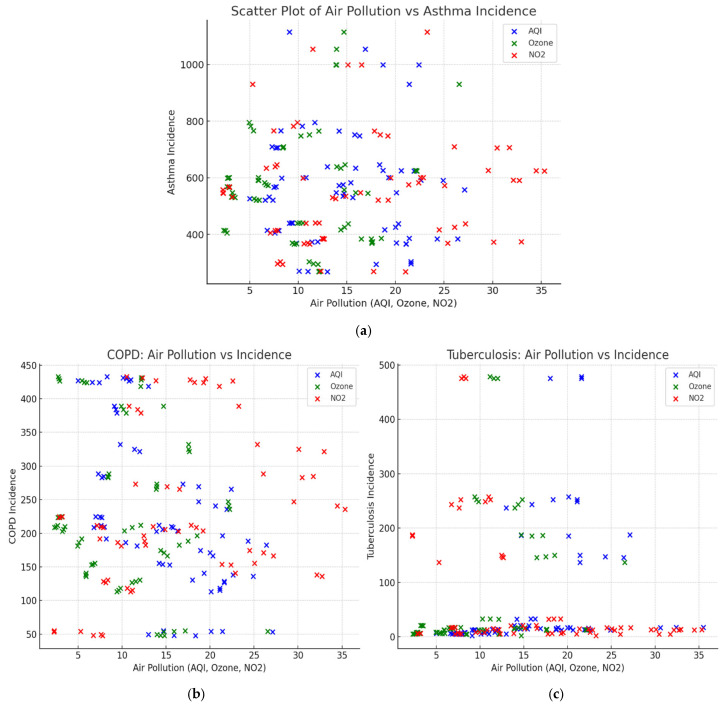
Scatter plots showing correlation between pollutants and disease incidence after controlling for covariates (**a**) Air pollution against asthma incidence (**b**) Air pollution against COPD incidence (**c**) Air pollution against tuberculosis incidence.

Figure 3 displays the scatter plots showing the relationships between three air pollutants (AQI, ozone, and NO_2_) and the incidence rates of three respiratory diseases (asthma, COPD, and tuberculosis). The scatter plot for asthma (Figure 3a) shows a broad distribution of cases across all pollutant levels. While there is no clear linear trend, some clustering occurs at moderate levels of ozone and NO_2_ (10–25), suggesting that pollutants may contribute to asthma but are not the sole determinants. Asthma incidence is influenced by a combination of environmental, genetic, and allergen exposure factors, which could explain the variability seen in the plot.

Figure 3b highlights COPD incidence against air pollutants. Unlike asthma, COPD shows a more defined positive correlation with ozone and NO_2_ levels, particularly at higher concentrations. This trend aligns with the established role of these pollutants in inducing chronic inflammation and airway damage, which are hallmark features of COPD pathophysiology. The relationship with AQI appears less pronounced, potentially because AQI is a composite measure, diluting the impact of specific pollutants like NO_2_ and ozone on COPD.

In Figure 3c, tuberculosis incidence remains low and scattered across pollutant levels, with no obvious pattern. This is consistent with tuberculosis being an infectious disease driven by factors like socioeconomic conditions and immune status, rather than direct environmental pollutants. While pollutants may indirectly weaken lung defenses, their role in driving tuberculosis is minimal compared to infections. Overall, these graphs highlight stronger associations between pollution and COPD, moderate links to asthma, and negligible influence on tuberculosis.

### 3.5. ANOVA Analysis

Table 7 shows the one-way ANOVA analysis; the analysis examined the variance in asthma, COPD, and tuberculosis incidence between high- and low-pollution periods, across all countries, for three key pollutants: AQI, ozone, and NO_2_.

For asthma, none of the pollution levels showed statistically significant variance. AQI levels (F = 1.45, *p* = 0.23), ozone levels (F = 0.001, *p* = 0.98), and NO_2_ levels (F = 1.92, *p* = 0.17) were not significantly associated with differences in asthma incidence between high- and low-pollution periods.

In contrast, COPD incidence showed significant variance with AQI (F = 42.84, *p* < 0.001) and NO_2_ levels (F = 15.32, *p* < 0.001), indicating that periods of high pollution were strongly associated with increased COPD rates. However, ozone levels did not significantly affect COPD incidence (F = 2.95, *p* = 0.09).

For tuberculosis, all three pollutants were significantly associated with differences in disease incidence between high- and low-pollution periods. AQI (F = 38.35, *p* < 0.001), NO_2_ (F = 20.09, *p* < 0.001), and ozone (F = 5.27, *p* = 0.02) all showed significant variance, suggesting that higher-pollution periods corresponded with increased tuberculosis incidence.

These findings demonstrate that while asthma incidence was not significantly affected by pollution levels, both COPD and tuberculosis showed strong associations with high-pollution periods, particularly for AQI and NO_2_. These findings highlight that while asthma incidence was not significantly affected by pollution levels, both COPD and tuberculosis showed strong associations with high-pollution periods, particularly for AQI and NO_2_.

**Figure 4:**
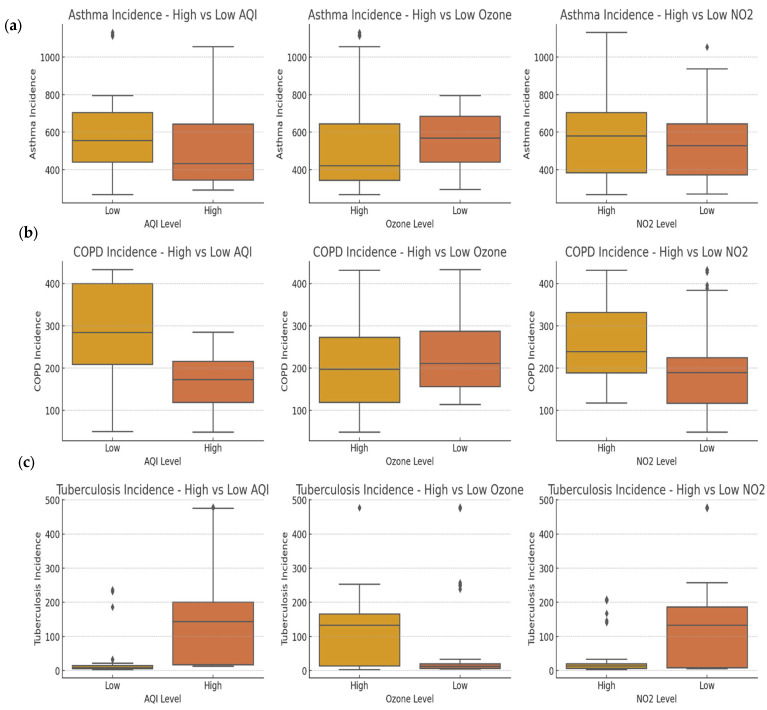
Comparison of disease incidence across high- and low-pollution periods using box plots, (**a**) Asthma incidence against AQI, ozone and NO_2_ level (**b**) COPD incidence against AQI, ozone and NO_2_ level (**c**) Tuberculosis incidence against AQI, ozone and NO_2_ level.

Figure 4 shows box plots that compare the incidence rates of asthma, COPD, and tuberculosis across high and low levels of air pollutants (AQI, ozone, and NO_2_). For asthma (Figure 4a), the data indicate minimal differences between high and low pollutant levels for AQI, ozone, and NO_2_. While the median values of asthma incidence are slightly elevated in the high-pollutant categories, the large interquartile ranges and overlapping whiskers suggest weak or inconsistent associations. This finding aligns with the multifactorial nature of asthma, where genetic predispositions, allergens, and environmental triggers collectively influence disease risk.

Figure 4b shows the box plots for COPD, which reveal more distinct differences between high- and low-pollutant categories. High AQI levels appear to correlate with lower COPD incidence, an unexpected finding possibly reflecting confounding factors like the composite nature of AQI or differing exposure sources. However, for ozone and NO_2_, the data indicate that high pollutant levels are associated with higher COPD incidence, as evidenced by elevated medians and wider distributions. This supports established research linking these pollutants to chronic airway inflammation and damage, which are key processes in COPD pathogenesis.

Figure 4c depicts tuberculosis incidence, which is consistently higher in high-pollutant categories for AQI, ozone, and NO_2_. Notably, while the median tuberculosis incidence remains low, the presence of outliers in the high-pollutant groups suggests that specific populations or regions with high exposure may experience greater vulnerability. This trend could reflect the indirect role of pollutants in weakening lung defenses, thus increasing susceptibility to infections. Overall, the data indicate strong pollutant links with COPD, potential outlier-driven associations with tuberculosis, and weaker relationships for asthma.

### 3.6. Discussion

This study explored the complex associations between air pollution and respiratory disease incidence across 27 countries, while also examining country-specific variations. This research highlights the crucial influence of environmental factors, as well as socioeconomic conditions, on the prevalence of respiratory diseases.

#### 3.6.1. Associations Between Air Pollution Markers and the Respiratory Disease Incidence Rates

The findings of this research reveal diverse associations between Air Quality Index (AQI), ambient ozone, and NO_2_ concentrations, and the incidence of respiratory diseases such as asthma, chronic obstructive pulmonary disease (COPD), and tuberculosis across all countries.

For asthma, the analysis revealed no significant correlations with AQI across all countries, and in fact, a significant negative correlation was found with AQI for COPD. This surprising result—indicating that poorer air quality may be associated with a lower prevalence of these conditions—may be attributed to potential underreporting or discrepancies in data from high-pollution areas. As AQI is a composite measure, it could mask the effects of specific pollutants, leading to an observed negative relationship with disease incidence. Furthermore, factors such as local health systems, access to healthcare, and socioeconomic determinants may contribute to the lack of a clear association [30]. However, when accounting for socioeconomic factors, a significant negative correlation between AQI and COPD remained, suggesting that the observed effects were driven by confounding variables rather than a direct pollutant–disease relationship.

In contrast, AQI exhibited a significant positive correlation with tuberculosis incidence across countries. This aligns with the hypothesis that air pollution could compromise immune function, increasing vulnerability to infections like tuberculosis. Similar findings have been observed in previous studies, where air pollution has been linked to respiratory infections by impairing lung function and weakening immune defenses [31,32]. The complex relationship between air pollution and tuberculosis incidence suggests that pollution may not directly cause tuberculosis, but could exacerbate the disease through weakened immune responses and heightened susceptibility in regions with elevated pollution levels.

Ambient ozone, in general, showed a negative correlation with asthma and COPD across all countries, but this relationship became insignificant after controlling for socioeconomic factors. Ozone is a known irritant that can contribute to airway inflammation, and it is plausible that in certain areas, the effects of ozone exposure are exacerbated by other environmental and social factors. However, after adjusting for confounders, the overall lack of significant findings suggests that the observed correlations between ozone and respiratory diseases may be largely influenced by socioeconomic factors.

The observed strong link between NO_2_ levels and COPD aligns with previous research, which highlights NO_2_ as a key contributor to lung inflammation and respiratory harm. NO_2_ exposure is known to exacerbate COPD symptoms by promoting chronic inflammation and damaging airway tissues [27,33]. This study found that higher NO_2_ levels were consistently associated with increased COPD incidence, confirming the harmful effects of NO_2_ on respiratory health. In contrast, the generally negative correlation between NO_2_ and tuberculosis incidence could reflect the complex dynamics of urbanization and healthcare access. Urban areas with higher NO_2_ levels may also have better healthcare infrastructure, more effective TB control programs, and improved living conditions, which could reduce the risk of tuberculosis despite higher levels of pollution [34,35]. This suggests that the relationship between NO_2_ and tuberculosis incidence is multifaceted and may depend on factors beyond pollution exposure alone.

#### 3.6.2. Regional Variability in the Impact of Air Pollution on Respiratory Disease Incidence

The relationship between air pollution and respiratory diseases varies significantly across countries, reflecting the complex interplay of environmental exposure, healthcare infrastructure, and socioeconomic conditions. While broad trends suggest that poor air quality increases respiratory disease incidence, regional disparities highlight the need to consider local factors when interpreting these associations. In Japan, AQI showed a strong positive correlation with tuberculosis, a pattern also observed in Brazil, Portugal, and Angola. This suggests that high pollution levels may weaken immune defenses, increasing susceptibility to TB. However, this association was not seen in India, despite its high TB burden and pollution levels. This discrepancy could be due to differences in disease surveillance and reporting. Japan has a highly structured TB control program that ensures accurate detection, whereas underreporting in India, particularly in rural areas, may obscure this relationship. Additionally, Japan’s aging population, concentrated in urban areas with higher pollution exposure, may contribute to increased TB susceptibility, whereas in India, malnutrition and co-infections play a more dominant role in TB transmission.

Variability in the association between ozone and COPD further underscores the role of local conditions. In Cambodia, Brazil, and South Africa, ozone levels were strongly correlated with COPD, likely due to widespread exposure to industrial emissions, biomass burning, and vehicle pollution. Limited healthcare access in these regions may exacerbate the impact of pollution on respiratory health. Conversely, countries such as Macedonia, Portugal, and Sweden showed a negative correlation, which could reflect stronger environmental regulations, lower exposure to extreme ozone levels, and better adaptation measures such as public health warnings on high-ozone days. Thailand presents an unexpected finding, where AQI was negatively correlated with COPD. This could be attributed to Thailand’s strict air pollution control measures in major cities, which may have led to better respiratory outcomes. Additionally, improved healthcare access in less polluted regions could lead to higher COPD detection rates in these areas, artificially creating a negative association.

A similar pattern was observed in Germany and Costa Rica, where COPD diagnosis may be more prevalent in regions with lower pollution due to greater healthcare availability and awareness. Ozone’s negative correlation with tuberculosis in Poland, Cambodia, Brazil, and Colombia raises questions about its potential antimicrobial properties. Some studies suggest that high ozone concentrations could reduce airborne pathogen viability, potentially lowering TB transmission rates in polluted urban areas [36,37]. Additionally, these countries have well-structured TB control programs that may counteract the expected negative health effects of pollution, leading to reduced TB incidence despite high ozone levels. These disparities emphasize the need for localized public health interventions. Countries with a strong AQI-TB correlation, such as Japan and Brazil, should integrate air pollution mitigation with TB prevention strategies. Nations with high ozone–COPD associations, like Cambodia and Brazil, should focus on reducing industrial and vehicular emissions while expanding healthcare access for COPD patients. In regions like Thailand, where pollution control policies appear effective, further efforts should ensure equitable access to respiratory healthcare in both urban and rural settings.

#### 3.6.3. Socioeconomic Factors: Influence on the Association Between Air Pollution and Respiratory Diseases

Socioeconomic factors like GDP per capita, tobacco smoking prevalence, and healthcare expenditure significantly influenced the associations between air pollutants and respiratory disease incidence. Higher GDP per capita showed a strong positive association with COPD, indicating that wealthier countries tend to report more cases, possibly due to better diagnostic practices or a longer life expectancy where COPD is more common. In contrast, GDP per capita was negatively associated with tuberculosis, suggesting that wealthier nations have better public health systems that can prevent or manage tuberculosis outbreaks. This finding aligns with previous research indicating that higher-income nations have more effective tuberculosis control measures, including vaccination programs, better access to healthcare, and improved living conditions consistent with previous studies [35,38]. These factors likely contribute to a reduced burden of tuberculosis in wealthier nations, despite the potential for increased exposure to pollution in urbanized areas.

Healthcare expenditure showed a positive correlation with asthma and COPD but a negative correlation with tuberculosis, reflecting better treatment and management for chronic conditions but effective preventive measures for infectious diseases like tuberculosis in countries with higher healthcare spending. Tobacco smoking prevalence, a well-known risk factor, was positively associated with COPD across all countries, highlighting the crucial role of smoking cessation in reducing COPD incidence [38]. However, its negative association with asthma may reflect the decreasing smoking rates in countries with higher asthma rates due to successful public health campaigns against smoking.

#### 3.6.4. Impact of Pollution Periods on Respiratory Diseases

The findings of this study demonstrate a clear association between periods of elevated pollution and increased incidence rates of COPD and tuberculosis, while asthma did not show a significant connection with these pollution periods. Specifically, elevated AQI and NO_2_ levels were strongly correlated with higher rates of COPD and tuberculosis, suggesting that periods of high pollution may exacerbate or trigger the onset of these respiratory conditions. This supports the hypothesis that pollution surges have a detrimental impact on respiratory health, particularly in vulnerable populations, where exposure to poor air quality can worsen existing conditions or lead to new diagnoses.

Previous research also supports the notion that short-term pollution spikes have a more pronounced effect on conditions like COPD and tuberculosis, which involve chronic inflammation and long-term damage to lung tissue [24,39]. The cumulative nature of COPD, which involves ongoing damage to the airways, makes individuals with this condition more susceptible to the harmful effects of elevated pollution. Similarly, tuberculosis, an infectious disease, can become more severe in individuals living in polluted environments, as air pollution may impair lung defenses and increase susceptibility to infections [3,7,8].

### 3.7. Limitations of the Study

Although this study gives important insights into the associations between air pollution and respiratory disease incidence rates, the cross-sectional design of this research limits its ability to draw causal inferences. Also, several confounders such as urbanization, occupational exposures, indoor air pollution, and genetic factors were not accounted for in the study.

### 3.8. Recommendations for Future Research

Future research should implement longitudinal designs to shed light on the causal relationships between air pollution exposure and the incidence of respiratory diseases over time, thereby facilitating a more comprehensive understanding of the long-term effects of pollution and the efficacy of pollution control measures. Additionally, increasing the temporal and geographic granularity such as monthly or seasonal data and conducting subnational analyses, could offer deeper insights into how short-term pollution changes and local conditions impact respiratory health. Accounting for a wider range of confounders, especially those excluded in this study, would also help isolate the specific effects of outdoor pollution. Examining these factors might facilitate future studies in understanding the relationship between air pollution and respiratory health, hence providing more robust evidence for successful public health interventions and policy formulation.

## 4. Conclusions

This study explored the associations between respiratory disease incidence, air pollution markers, and socioeconomic factors. The findings provide evidence that the incidence of respiratory diseases is influenced by a combination of air pollution levels and socioeconomic conditions, highlighting that a multifactorial approach is needed to mitigate respiratory disease incidence.

Policymakers must enforce stricter emission regulations, promote cleaner energy solutions, and invest in sustainable transportation options to reduce air pollution levels. In addition to these traditional regulatory measures, real-time pollution monitoring, smart infrastructure, and incentives for cleaner technologies should be considered. Governments should integrate pollution-responsive urban planning, including air-filtering buildings and green ventilation corridors, to reduce exposure in high-risk areas.

In countries with lower economic resources and high smoking prevalence, strengthening healthcare infrastructure is essential for managing respiratory conditions more effectively. Early detection and management of pollution-related diseases must be prioritized. Decentralizing respiratory care through mobile clinics and expanding insurance coverage for pollution-induced illnesses would improve access to treatment. Workplace screening for respiratory diseases in high-risk industries and subsidized access to protective measures (such as indoor air purification systems) could further reduce health disparities. Additionally, public health initiatives aimed at reducing tobacco consumption should work in tandem with pollution reduction efforts, as smoking intensifies the harmful effects of poor air quality. Tobacco control policies require a shift from traditional taxation to integrated cessation programs within primary healthcare services. Trade restrictions on low-cost, high-toxicity tobacco products and economic incentives for transitioning workers away from tobacco production could strengthen public health efforts.

Material science innovations should also be leveraged. The adoption of pollution-absorbing building materials, photocatalytic coatings, and genetically enhanced air-purifying plants could transform urban spaces into active pollution mitigators. Governments should support research into affordable, scalable solutions that can be implemented in both high- and low-income regions

Addressing these interconnected challenges can significantly improve respiratory health outcomes and reduce the overall disease burden. In conclusion, both air pollution and healthcare gaps must be addressed to ensure the global burden of respiratory disease is reduced. Governments and health organizations must act swiftly to implement policies that improve air quality and strengthen healthcare systems, particularly in vulnerable, low-income regions.

## Figures and Tables

**Table 1:** Summary statistics for key variables.

Variable	Mean	Std Dev	Min	Max	25th Percentile	75th Percentile
AQI	21.68	19.09	5.00	97.00	9.38	32.00
Ozone (ppb)	14.73	12.14	2.33	49.16	5.93	19.69
NO_2_ (ppb)	15.70	8.70	2.22	35.33	8.11	23.13
Asthma (per 100k)	558.40	220.84	266.37	1132.42	373.46	665.78
COPD (per 100k)	225.99	112.81	47.99	450.57	140.91	322.37
Tuberculosis (per 100k)	79.82	112.93	2.37	452.63	8.13	96.97
GDP per capita (USD)	20,172.06	20,648.17	758.30	71,056.00	2519.87	27,576.82
Health Expenditure (USD)	2191.69	2811.98	23.85	12,051.10	120.43	3030.46

**Table 2:** Pearson correlation result across all countries.

Pollutant	Disease	Correlation	*p*-Value
AQI	Asthma	−0.31975	<0.001
AQI	COPD	−0.34001	<0.001
AQI	Tuberculosis	0.376927	<0.001
OZONE	Asthma	−0.25681	0.007297
OZONE	COPD	−0.26938	0.004815
OZONE	Tuberculosis	0.376529	<0.001
NO_2_	Asthma	0.026574	0.78485
NO_2_	COPD	0.332522	<0.001
NO_2_	Tuberculosis	−0.40483	<0.001

**Table 3:** Significant results of country-by-country Pearson correlation.

Country	Pollutant	Disease	Correlation	*p*-Value
Japan	AQI	Tuberculosis	0.986431	0.013569
Poland	OZONE	COPD	0.977646	0.022354
Poland	OZONE	Tuberculosis	−0.98662	0.013381
Thailand	AQI	COPD	−0.9902	0.009804
Thailand	OZONE	COPD	0.95613	0.04387
Macedonia	AQI	COPD	−0.95414	0.045863
Macedonia	AQI	Tuberculosis	0.974516	0.025484
Macedonia	OZONE	COPD	−0.99894	0.001062
Germany	AQI	COPD	−0.98862	0.011379
Cambodia	OZONE	COPD	0.997751	0.002249
Cambodia	OZONE	Tuberculosis	−0.96493	0.035073
Costa Rica	AQI	Asthma	0.985665	0.014335
Costa Rica	AQI	COPD	−0.9583	0.041697
Costa Rica	OZONE	Asthma	−0.99545	0.004553
Costa Rica	OZONE	COPD	0.980057	0.019943
New Zealand	AQI	Tuberculosis	−0.99361	0.006389
New Zealand	OZONE	Tuberculosis	0.991269	0.008731
Portugal	AQI	Asthma	0.995486	0.004514
Portugal	AQI	Tuberculosis	0.964202	0.035798
Portugal	OZONE	COPD	−0.9902	0.009804
Sweden	OZONE	COPD	−0.961	0.039002
Brazil	AQI	COPD	−0.95032	0.049679
Brazil	AQI	Tuberculosis	0.982348	0.017652
Brazil	OZONE	COPD	0.991433	0.008567
Brazil	OZONE	Tuberculosis	−0.99982	0.000178
Mexico	OZONE	Asthma	0.985579	0.014421
Mexico	OZONE	COPD	−0.97464	0.025362
Chile	AQI	Asthma	−0.95829	0.041708
Colombia	OZONE	Asthma	−0.96736	0.032645
Colombia	OZONE	COPD	0.998609	0.001391
Colombia	OZONE	Tuberculosis	−0.99968	0.00032
United States	OZONE	COPD	−0.99959	0.00041
United States	OZONE	Tuberculosis	−0.98343	0.01657
Argentina	AQI	Tuberculosis	0.953342	0.046658
Argentina	OZONE	Asthma	−0.96261	0.037394
Argentina	OZONE	COPD	0.972659	0.027341
Argentina	OZONE	Tuberculosis	−0.95793	0.042072
Turkey	OZONE	COPD	−0.99576	0.004239
India	OZONE	COPD	0.996541	0.003459
India	OZONE	Tuberculosis	0.983482	0.016518
Ethiopia	OZONE	COPD	0.99897	0.00103
Pakistan	OZONE	Tuberculosis	−0.99273	0.007273
Pakistan	NO2	COPD	−0.95445	0.045552
South Africa	OZONE	Asthma	−0.95643	0.043567
South Africa	OZONE	COPD	0.992937	0.007063
Nigeria	OZONE	COPD	0.999815	0.000185
Nigeria	OZONE	Tuberculosis	−0.99837	0.00163
Bangladesh	AQI	Asthma	0.974085	0.025915
Bangladesh	OZONE	COPD	0.998054	0.001946
Bangladesh	OZONE	Tuberculosis	−0.96288	0.037117
Angola	AQI	COPD	−0.99351	0.00649
Angola	AQI	Tuberculosis	0.993709	0.006291
Angola	OZONE	Tuberculosis	0.950886	0.049114

**Table 4:** Correlation between socioeconomic factors and disease incidence across all countries.

Socioeconomic Factor	Disease	Coeff (*p*-Value)
GDP per capita	Asthma	0.18 (0.12)
GDP per capita	COPD	0.77 (<0.001)
GDP per capita	Tuberculosis	−0.55 (<0.001)
Tobacco use	Asthma	−0.29 (0.01)
Tobacco use	COPD	0.31 (0.005)
Tobacco use	Tuberculosis	0.01 (0.95)
Health expenditure	Asthma	0.26 (0.02)
Health expenditure	COPD	0.74 (<0.001)
Health expenditure	Tuberculosis	−0.48 (<0.001)

**Table 5:** Significant results for country-by-country correlation between socioeconomic factors and disease incidence.

Country	Confounder	Disease	Corr (*p*-Value)
Turkey	GDP/capita	COPD	−0.99 (0.04)
Angola	GDP/capita	COPD	−0.99 (0.007)
Ethiopia	GDP/capita	COPD	0.99 (0.01)
Chile	GDP/capita	COPD	−0.99 (0.05)
Australia	GDP/capita	Tuberculosis	0.99 (0.02)
New Zealand	GDP/capita	Tuberculosis	−0.99 (0.001)
Bangladesh	GDP/capita	Tuberculosis	−0.99 (0.05)
Australia	Tobacco use	Asthma	0.99 (0.04)
Poland	Tobacco use	Asthma	−0.99 (0.003)
Sweden	Tobacco use	Asthma	−0.99 (0.009)
New Zealand	Tobacco use	Asthma	0.99 (0.03)
India	Health expenditure	COPD	0.99 (0.01)
Ethiopia	Health expenditure	COPD	0.99 (0.03)
Thailand	Health expenditure	COPD	0.99 (0.04)
Bangladesh	Health expenditure	COPD	0.99 (0.04)
Bangladesh	Health expenditure	Tuberculosis	−0.99 (0.02)

**Table 6:** Multivariate regression analysis after controlling for covariates.

**Pollution**	**Asthma** **β (*p*-Value)**	**COPD** **β (*p*-Value)**	**Tuberculosis** **β (*p*-Value)**
AQI	−4.33 (0.56)	−7.34 (0.0087)	0.29 (0.907)
Ozone	2.997 (0.552)	2.05 (0.258)	4.31 (0.0673)
NO2	3.937 (0.211)	1.26 (0.254)	−5.10 (<0.001)

**Table 7:** Analysis of variance in disease incidence during low- and high-pollution periods.

Disease	Pollution Level	F-Statistic (*p*-Value)
Asthma	AQI_level	1.45 (0.23)
Asthma	OZONE_level	0.001 (0.98)
Asthma	NO_2__level	1.92 (0.17)
COPD	AQI_level	42.84 (<0.001)
COPD	OZONE_level	2.95 (0.09)
COPD	NO2_level	15.32 (<0.001)
Tuberculosis	AQI_level	38.35 (<0.001)
Tuberculosis	OZONE_level	5.27 (0.02)
Tuberculosis	NO_2__level	20.09 (<0.001)

## Data Availability

The links for the raw data required to reproduce these findings are provided in Section 2.3.

## Appendix A

List of countries used in this study.

1. Japan
2. Australia
3. Poland
4. Thailand
5. Macedonia
6. Germany
7. Cambodia
8. Costa Rica
9. New Zealand
10. Canada
11. Portugal
12. Sweden
13. Brazil
14. Mexico
15. Chile
16. Colombia
17. United Kingdom
18. United States
19. Argentina
20. Turkey
21. India
22. Ethiopia
23. Pakistan
24. South Africa
25. Nigeria
26. Bangladesh
27. Angola
